# Does the intensive grazing and aridity change the relations between the dominant shrub *Artemisia kopetdaghensis* and plants under its canopies?

**DOI:** 10.1002/ece3.8124

**Published:** 2021-09-22

**Authors:** Soroor Rahmanian, Hamid Ejtehadi, Mohammad Farzam, Martin Hejda, Farshid Memariani, Petr Pyšek

**Affiliations:** ^1^ Department of Biology Faculty of Sciences Ferdowsi University of Mashhad Mashhad Iran; ^2^ Department of Range and Watershed Management Faculty of Natural Resources and Environment Ferdowsi University of Mashhad Mashhad Iran; ^3^ Institute of Botany Department of Invasion Ecology The Czech Academy of Sciences Průhonice Czech Republic; ^4^ Department of Botany Research Center for Plant Sciences Ferdowsi University of Mashhad Mashhad Iran

**Keywords:** *Artemisia kopetdaghensis*, CSR plant strategies, facilitation, herbivory, plant–plant interactions

## Abstract

The interspecific plant interactions along grazing and aridity stress gradients represent a major research issue in plant ecology. However, the combined effects of these two factors on plant–plant interactions have been poorly studied in the northeast of Iran. To fill this knowledge gap, 144 plots were established in 12 study sites with different grazing intensities (high vs. low) and climatic characteristics (arid vs. semiarid) in northeastern Iran. A dominant shrub, *Artemisia kopetdaghensis*, was selected as the model species. Further, we studied changes in plant life strategies along the combined grazing and aridity stress gradients. In this study, we used relative interaction indices calculated for species richness, Shannon diversity, and species cover to determine plant–plant interactions using linear mixed‐effect models (LMM). The indicator species analysis was used to identify the indicator species for the undercanopy of shrub and for the adjacent open areas. The combined effects of grazing and aridity affected the plant–plant interactions and plant life strategies (CSR) of indicator species. *A. kopetdaghensis* showed the highest facilitation effect under high stress conditions (high grazing, high aridity), which turned into competition under the low stress conditions (low grazing, low aridity). In the arid region, the canopy of the shrub protected ruderals, annual forbs, and grasses in both high and low grazing intensities. In the semiarid region and high grazing intensity (low aridity/high grazing), the shrubs protected mostly perennial forbs with C‐strategy. Our findings highlight the importance of context‐dependent shrub management to restore the vegetation damaged by the intensive grazing.

## Graphical abstract

The manuscript is based on a field study, aiming to compare the canopy effects of an unpalatable dominant shrub (*Artemisia kopetdaghensis*) in the conditions of biotic stress, represented by intensive grazing and abiotic stress, represented by severe aridity. Our results bring new evidence to the current debate on the relative importance and characteristics of plant–plant interactions along the gradients of environmental stress. We detected the facilitative effects of the dominant shrub on species diversity in the presence of both grazing and aridity. However, the facilitative effects turned into competition in the conditions of less severe aridity in the semiarid region.

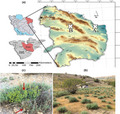

## 1 INTRODUCTION

Species interactions are known as a key driver of the plant community structure, biodiversity, ecosystem function, and dynamics (Brooker et al., 2008; Callaway et al., 2005; Jankju, 2008, 2013). However, the outcome of plant–plant interactions may vary, ranging from competition to facilitation, depending on environmental severity (Brooker & Callaghan, 1998; Grime, 1977) and coexisting species (Armas et al., 2011; Pugnaire et al., 2011). Interactions among plants may reduce the extreme effects of abiotic and biotic stress such as aridity (López et al., 2016) and intensive grazing (Holmgren & Scheffer, 2010; Smit et al., 2009; Smit et al., 2007; Soliveres et al., 2011) by creating suitable microhabitats for drought‐ or grazing‐intolerant species (Bruno et al., 2003; Farzam & Ejtehadi, 2017).

Grazing is a key biotic stress in dry rangelands due to its extensive application and its potential to change the community structure and species composition, and to degrade the ecosystem services (Diaz et al., 2007; Jankju, 2016, Kéfi et al., 2007; Li et al., 2013, Rahmanian et al., 2019, 2020). Previous studies examining the herbivores as main drivers have reported that unattractive, toxic, or thorny plants may induce positive indirect (i.e., grazer‐mediated) effects on palatable herbs, shrubs, or trees (Bakker et al., 2004; Callaway et al., 2005; Smit et al., 2006). Grazing may affect the plant–plant interactions as well (Soliveres et al., 2011). The result of these interactions will be highly dependent on the ability of the nurse plant to moderate the effects of herbivores and on the tolerance of the facilitated species to grazing (Baraza et al., 2006; Vandenberghe et al., 2009). Further, nurse plants may protect the neighbors against herbivory and enhance their survival by increasing resource availability (Acuña‐Rodríguez et al., 2006; Rand, 2004).

The CRS strategy, distinguishing between the competitors, ruderals, and stress tolerators (CSR—Grime, 1979), provides a detailed view on the characteristics of indicator species for different types of vegetation, that is, across plant growth forms and differences in the intensity of environmental stress and disturbance and/or grazing (Grime, 1977; Hodgson et al., 1999).

Previous researches have explored the effects of livestock grazing and climate on the relationships between plants (Berdugo et al., 2019; Metz & Tielbörger, 2016), but its effects on plant–plant interactions have rarely been addressed (but see Filazzola et al., 2017; Soliveres et al., 2011; Verwijmeren et al., 2014). However, previous studies have reported varying effects of grazing on plant–plant interactions. For instance, Soliveres et al. (2011) showed that rabbit grazing caused positive interactions between the bunch grass (*Stipa tenacissima*) and saplings of the shrub (*Retama sphaerocarpa*) during winter and autumn. However, because of higher grazing intensity in the summer, the interspecific interactions shifted to neutral. Similarly, Holthuijzen and Veblen (2015) found that positive interactions between *Artemisia tridentata* ssp. *wyomingensis* and *Poa secunda* decreased with increasing grazing intensity in the arid region because grazing reduced the productivity during the drought periods more intensively. This may result in the absence of positive interactions between plants due to different stressors (Smit et al., 2009; Verwijmeren et al., 2014; Michalet et al., 2014). On the contrary, Noumi et al. (2016) showed that suppressive effects of shrubs on *Acacia tortilis* seedlings shifted to positive with increasing grazing stress. Therefore, an increase in facilitation due to the combination of these two stressors can be expected. This research aims at investigating plant–plant interactions, accounting for the combined effects of grazing and aridity.

The selected dominant species, *Artemisia kopetdaghensis*, is an aromatic shrub that is widely distributed, ranging from warm and arid to cold and semiarid steppes of northeast Iran (180–400 mm) and parts of Turkmenistan (Memariani, 2016). We used *A. kopetdaghensis* and its understory plants as a model system to study the combined effects of grazing and climate (arid region: high/low grazing, semiarid region: high/low grazing). Our aim was to answer the following questions: (a) What are the dominant interactions between *A. kopetdaghensis* as a target species and its surrounding understory herbs? (b) Are the relations between *A. kopetdaghensis* and plants in its surroundings affected by aridity and intensive grazing? And (c) do the combined effects of grazing and aridity differ between various plant life strategies?

## 2 METHODS

### 2.1 Study area

We selected two sites along the precipitation gradient in northeastern Iran in the Khorassan‐Kopet Dagh floristic province of the Irano‐Turanian region, located between 35°43′–36°44′N and 58°40′−60°27′E. Based on meteorological data, Khajeh Kalat has an arid climate and Baharkish has a semiarid climate, expressed by De Martonne aridity index (see Table 1 for more details). The mean annual precipitation (20‐year mean) is 255 mm in Khajeh Kalat and 385 in Baharkish. *Artemisia kopetdaghensis* Krasch.M. Pop. & Linecz ex Poljak was the dominant native shrub species in both sites. *Artemisia* species have been documented to facilitate common annual and perennial forbs (Reisner et al., 2015) by creating suitable microclimate, reducing evapotranspiration (e.g., Holthuijzen & Veblen, 2015), mediating soil temperatures (Davies et al., 2007), raising soil water content via hydraulic lift (e.g., Holthuijzen & Veblen, 2015), and accumulating soil nutrients (Cardon et al., 2013).

**TABLE 1:** Basic characteristics and grazing history of the arid and semiarid regions in northeastern Iran

Region	Khajeh Kalat 35°43′–35°50′N, 60°27′–60°34′E	Baharkish 36°44′–36°42′N, 58°40′–58°36′E
Climate classification (De Martonne)	Arid	Semiarid
Mean annual precipitation (mm)	255	385
Mean annual temperature	17.9	13
De Martonne Index	9	15.5
Elevation (m)	630–810	1,580–2,390

Management	Grazing area	Area protected from grazing	Grazing area	Area protected from grazing
Grazing history	Seasonal‐free ranging	protected in the last 35 years, occasional light grazing in some years	Seasonal‐free ranging	Protected in the last 35 years, occasional light grazing in some years
Grazing type	Seasonal, 20 March–10 May	Seasonal, 20 March–10 May	Seasonal, 20 May–23 July	Seasonal, 20 May–23 July
Grazers type	Sheep (90%), goat (10%)	Sheep (90%), goat (10%)	Sheep (90%), Goat (10%)	Sheep (90%), goat (10%)
Grazers density	3 AUM/ha	0–0.5 AUM/ha	2 AUM/ha	0–0.5 AUM/ha

Abbreviation: AUM, animal unit month.

### 2.2 Sampling design

**FIGURE 1:**
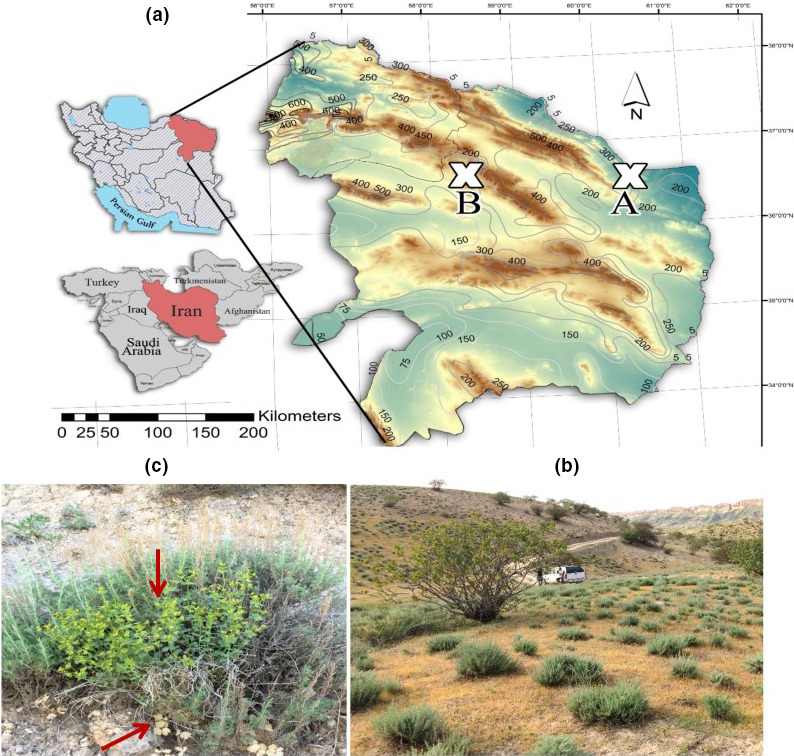
(a) Map of the study area in NE Iran, showing the Khajeh Kalat as an arid region, with ~255 mm of annual precipitation and Baharkish rangeland as a semiarid region, with ~385 mm of annual precipitation, (b) natural habitat with *Artemisia kopetdaghensis* as a dominant species, and (c) plant communities around *Artemisia kopetdaghensis*, the arrows point to other species under the canopy of *A. kopetdaghensis*

The two studied regions were 1,600 ha and 1,035 ha in size for the arid and semiarid regions, respectively. The HG and LG sites were of similar size in both climatic regions. The distance between individual sampling areas within each climatic region was less than one kilometer. The HG and pairwise LG sites were relatively homogenous in terms of topography, land use, and vegetation, and the only substantial difference between the paired HG and LG sites was in their grazing intensities. The LG sites were located within fences that have prevented grazing for around 35 years, whereas HG sites were open and therefore have suffered from long‐term overgrazing. Each plot was characterized by geographic coordinates and altitude. In 2017, the number of individuals and percentage cover of all vascular plant species were recorded between April and June, when the growing season peaks in this region (Figure 1).

The decision about the grazing status of the sites (high grazing intensity vs. occasional/low grazing intensity) was based on the median number of dung droppings (Marques et al., 2001): 55.3 dung droppings per square meter in the HG and 6.2 in the LG sites, and also on the width of the microterrace livestock paths in a horizontal way (0.27 ± 0.09 m for the HG site and 0.04 ± 0.03 m for the LG site) (see more information on the grazing history in Table 1).

**FIGURE 2:**
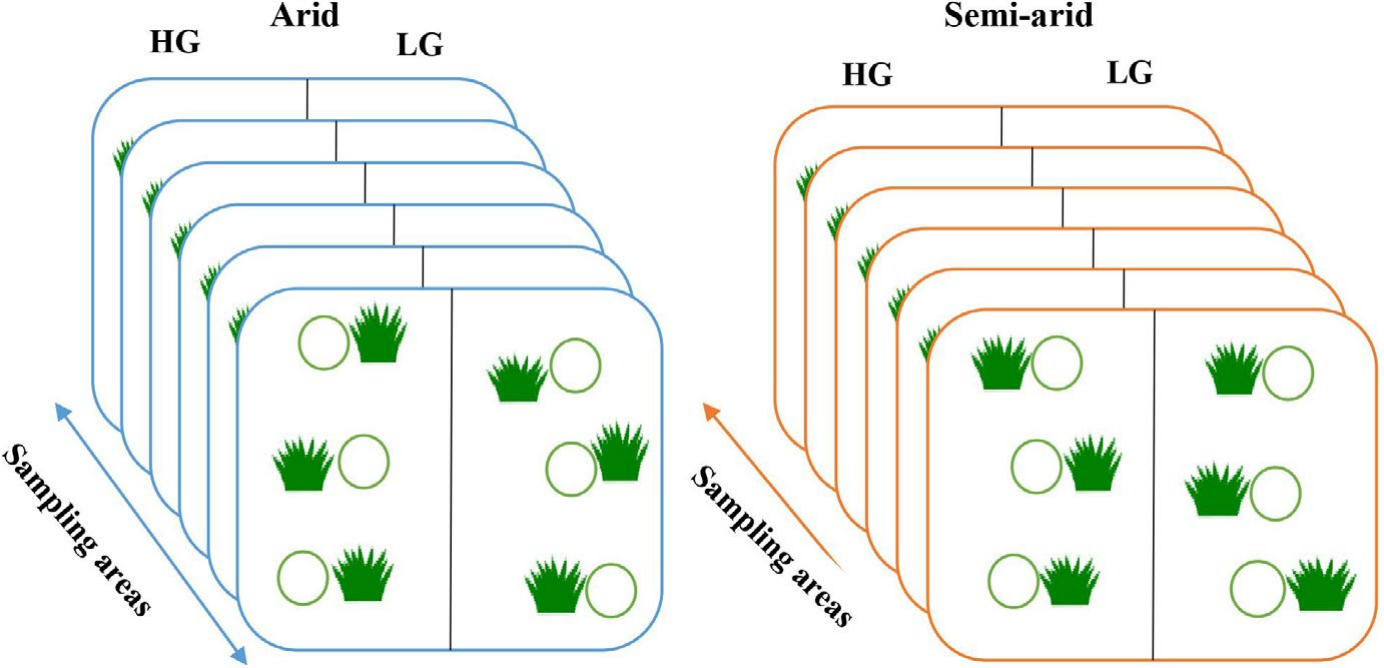
Graphical scheme of the sampling design. There are two studied regions (arid and semiarid), each containing six sampling areas with a high‐grazed and a low‐grazed site in each, arranged in a pairwise way (hereafter referred to as HG and LG sites). Three plots under the *Artemisia kopetdaghensis* shrubs and three adjacent plots outside the canopy of *A. kopetdaghensis* were sampled in each HG and LG site

The sampling design was arranged in a hierarchical way: In each of the two climatic regions (arid and semiarid), we selected six sampling areas, with a high‐grazed and a low‐grazed site in each sampling area, arranged in a pairwise way (hereafter referred to as HG and LG sites). Then, we sampled three plots under the *A. kopetdaghensis* shrubs and three adjacent plots outside the canopy of *A. kopetdaghensis* (hereafter referred to as undercanopy and open plots) in each HG and LG site (Soliveres et al., 2014). Altogether, 144 plots were sampled: 2 climatic regions, 6 sampling areas in each climatic region, one pairwise HG and one LG site in each sampling area, and 6 plots (3 undercanopy and 3 in the open) in each HG or LG sites (see Figure 2). We recorded the numbers of individuals of all vascular plant species and their percentage covers and then calculated the Shannon index of species diversity (H = −∑ pi ln pi) for each plot (Shannon, 1948), pi is the proportion (n/N) of individuals of one particular species (n) divided by the total number of individuals (N).

To obtain comparable samples in the surrounding “open” plots (outside the canopy of *A. kopetdaghensis*), matching the size of each sampled *A. kopetdaghensis* canopy, we sampled at randomly selected paired points, located ~1 m away from the canopy edge of each sampled *A. kopetdaghensis* shrub. When the size of *A. kopetdaghensis* was not measured, a wire loop was shaped to match the size of the sampled *A. kopetdaghensis* canopy plot and then used to define the size of the patch sampled in the “open” plot (Farzam & Ejtehadi, 2017). In addition, percentage covers of all vascular plants in plots in these open areas were recorded and identified to the species level.

### 2.3 Statistical analyses

Relative interaction intensity (RII) was used to assess the effect of shrubs on undercanopy vegetation (Armas et al., 2004) and was calculated based on the cover, richness, and diversity (expressed as Shannon index) of undercanopy vegetation: RII = (value under shrub – value in the open)/(value under shrub + value open). Samples were paired between each *A. kopetdaghensis* shrub and its neighboring open plot. RII was used as an indicator of the facilitation by the target shrub, based on the performance of undercanopy plants. The interaction index has defined limits [−1, +1], with positive values indicating facilitation and negative values indicating competition.

The differences in RII indices for species richness, cover, and diversity between the HG and LG sites and between the arid and semiarid regions were tested using linear mixed‐effect models, with “sampling areas” as a random effect, “climatic region” and “grazing” as fixed effects, and RII‐based richness (RII‐Richness), cover (RII‐Cover), and Shannon H (RII‐Shannon diversity) as response variables. All univariate analyses were performed in the R software, using the NLME package. The script for the model testing the interaction between “climate” and “grazing” was “lme (Relative interaction intensity~climatic region * grazing, random = ~1|sampling area).” The normality of the input data was assessed based on the Shapiro–Wilk tests, and the normality of residuals was checked visually, by plotting the observed values against the fitted values.

Further, we used the method of indicator species analysis to reveal the preference of individual species for the HG versus LG sites in both the arid and semiarid climatic regions. With this approach, we could determine the indicator species sensitive or resistant to high grazing intensity in two different climatic regions. Indicator species analysis has two main components: (a) recorded on either HG or LG sites only (exclusivity); and (b) recorded on all samples of either the HG or LG group (fidelity). The indicator value index was assigned to all species, identifying species with the highest association values. The permutation tests (999 permutations) were used to estimate the statistical significance of individual species' indicator values (Dufrêne & Legendre, 1997). The indicator species analyses were performed using the “indicspecies” package of the R software (R Development Core Team, 2013).

We also calculated the values for CSR plant strategies for all indicator species and for *A. kopetdaghensis*, following Pierce et al. (2017), based on the following traits: specific leaf area (SLA), leaf dry matter content (LDMC) and leaf area (LA). We collected the leaves from robust and well‐grown plants. Leaf material was collected from 10 individuals of each species (Behroozian et al., 2020), packed in moist paper bags, sealed in plastic bags and stored in a thermal box until storage at 4℃ for 12–24 hr. Depending on the size of leaves, 2–10 undamaged, fully expanded young leaves (including the petiole) were measured per individual. We determined the leaf area using a digital scanner and Leaf Area Measurement v1.3 software (Andrew Askew, University of Sheffield, UK). Turgid leaf fresh weight (LFW) was obtained from saturated leaves, and leaf dry weight was determined after drying for 72 hr in an oven at 70℃. For CSR strategy analysis, values of LA, SLA and LDMC were inserted into the “StrateFy” spreadsheet 3 to calculate C, S, and R percentages for each species (Pierce et al., 2017).

## 3 RESULTS

### 3.1 The effect of climate and grazing interaction on plant–plant relations

We found significant effects of both grazing and aridity on plant–plant interactions, expressed by the RII indices. In particular, the RII indices for species richness, cover, and Shannon diversity were all positive in high aridity/high grazing conditions. The RII values were negative for species richness, Shannon diversity, and cover in the low aridity/low grazing conditions and also for Shannon diversity in the low aridity/high grazing (Table 2).

**TABLE 2:** Results of linear mixed‐effect models, testing the effects of climate, grazing, and their interactions on RII‐Shannon, RII‐Richness, and RII‐Cover

	Climate			Grazing			Climate × grazing		
	*df*	*F*	*p*	*df*	*F*	*p*	*df*	*F*	*p*
RII‐Cover	1	17.46	<.001***	1	13	.0006***	2	15.6	<.0001***
RII‐Richness	1	9.56	.01**	1	5.07	.02*	2	4.88	.01**
RII‐Shannon	1	7	.02*	1	3.62	.06	2	3.87	.02*

***p < .001, **p < .01, *p < .05, and no asterisk (*p* < .1).

### 3.2 Interaction intensity along the stress gradient

**FIGURE 3:**
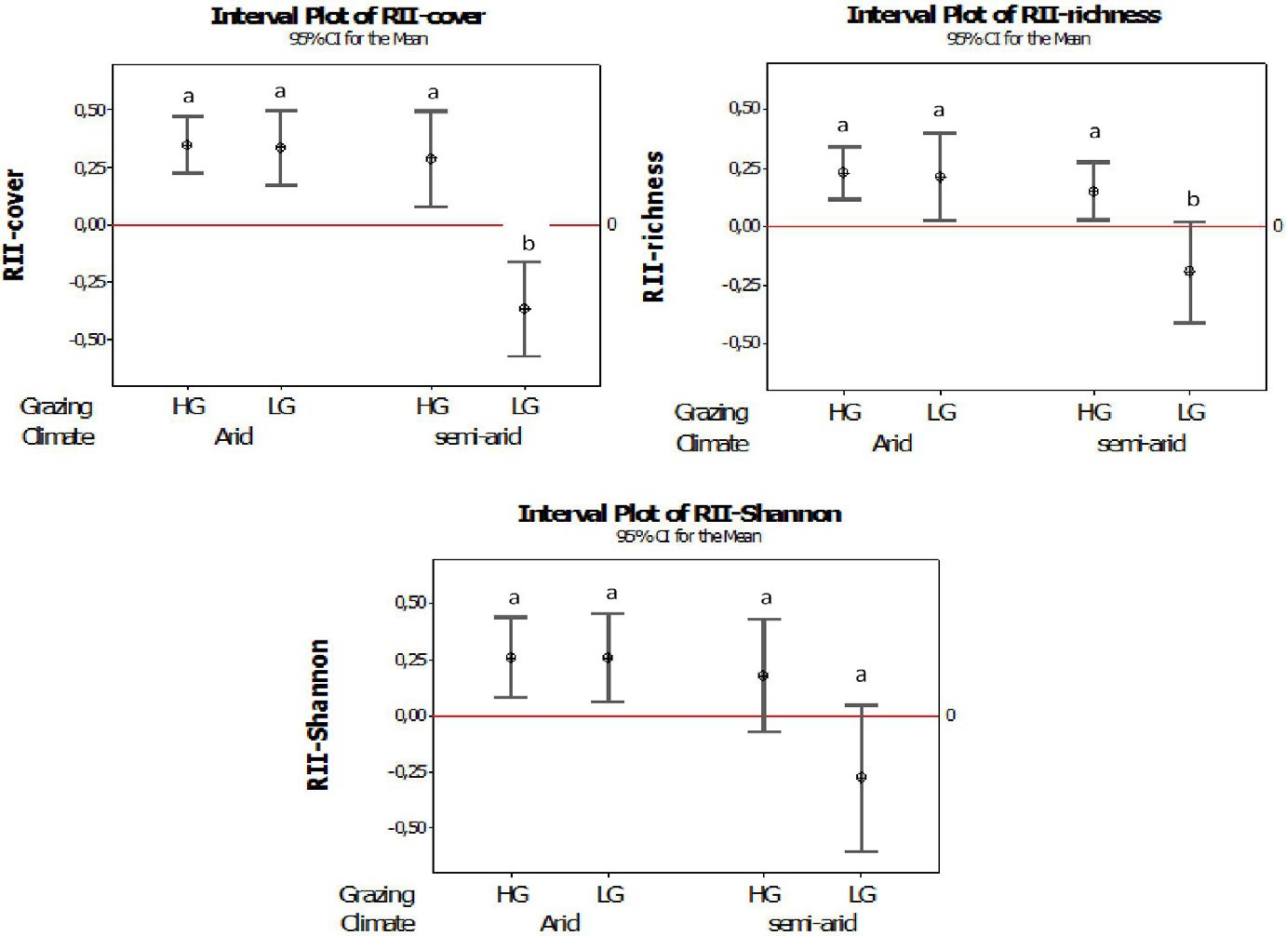
Comparisons of relative interaction indices (RII‐Richness, RII‐Cover, and RII‐Shannon diversity) of *Artemisia kopetdaghensis* between the HG and LG sites in the arid and semiarid regions

For all three indices (cover, richness, and Shannon diversity), RII was higher in the arid than in the semiarid climatic region (Figure 3). The RII indices for species' cover, species' richness, and Shannon diversity were all positive on both the LG and HG sites in the arid region, indicating a facilitative effect of the target shrub, *Artemisia kopetdaghensis* (Figure 3; Appendix A). However, the response of RII to the grazing intensity varied with climatic conditions. A significant facilitation (expressed by the positive RII values) was recorded in the semiarid region for species' cover, richness, and Shannon diversity, but only on the HG sites. The RII values for species' covers, richness, and Shannon diversity were negative for the LG sites in the semiarid region, indicating competition rather than facilitation by the dominant shrub (Figure 3; Appendix A).

### 3.3 Plant strategies and indicator species

The values of the CSR plant life strategies showed that *A. kopetdaghensis* was S‐selected in all combinations of grazing and aridity levels. Stress‐tolerant species were dominant under the shrub's canopy in both the high‐stress sites (high aridity/high grazing) and the low‐stress sites (low aridity/low grazing), that is, *Poa bulbosa* L., *Salsola dendroides* Pall., *Achillea biebersteini* Afan., and *Eremurus stenophyllus* (Boiss & Buhse) Baker. However, the stress‐tolerant species (S) were replaced by ruderals (R‐selected, i.e., *Alyssum desertorum* Stapf, *Astragalus filicaulis* Kar. & Kir., *Callipeltis cucullaria* (L.) Stev., *Galium tricornutum* Dandy) and competitors (C‐selected, i.e., *Cirsium bornmuelleri* Sint. ex Bornm., *Prunus pseudoprostrata* (Pojark.) Rech.f, *Thalictrum sultanabadense* Stapf; Table 3), respectively, on the sites with intermediate stress levels: low intensity of livestock grazing or aridity (high aridity/low grazing and low aridity/high grazing).

**TABLE 3:** List of indicator species found under *A. kopetdaghensis* canopy and on open plots of the HG and LG sites in the arid and semiarid regions, showing the exclusivity and fidelity of indicator species

Species of the undercanopy plots	Semiarid LG	Semiarid HG	Arid LG	Arid HG	Life cycle	CSR
*Achillea biebersteini* Afan.	0.045*				A	S
*Alyssum desertorum* Stapf			0.002**		A	R/SR
*Astragalus* (*Caprini*) *citrinus* Bunge		0.01*			P	S
*Astragalus filicaulis* Kar. & Kir.			0.002**		A	R/CSR
*Astragalus oxyglottis* M. Bieb.		0.01*			A	S/SR
*Bromus danthoniae* Trin.			0.03*		A	S
*Bromus tectorum* L.	0.005**				A	S/SR
*Callipeltis cucullaria* (L.) Stev.			0.04*		A	R
*Cirsium bornmuelleri* Sint. ex Bornm.		0.01*			P	CS
*Elymus hispidus *(Opiz) Melderi		0.001***			P	CSR
*Eremurus stenophyllus* (Boiss & Buhse) Baker	0.005**				P	S
*Galium tricornutum* Dandy			0.001***		A	R
*Lactuca orientalis* Boiss.	0.046*				P	CR
*Poa bulbosa* L.				0.006**	P	SR
*Prunus pseudoprostrata* (Pojark.) Rech.f		0.01*			P	S
*Salsola dendroides* Pall.				0.01*	P	SR
*Thalictrum sultanabadense* Stapf		0.005**			P	CSR
Species of the open plots	**Semiarid LG**	**Semiarid HG**	**Arid LG**	**Arid HG**	**Life cycle**	**CSR**
*Achillea biebersteini* Afan.	0.04*				A	S
*Aegilops triuncialis* L.	0.004**				A	SR
*Poa bulbosa* L.				0.01*	P	SR

Note

Significances refer to indicator values (exclusivity × fidelity) (*p < .05, **p < .01, and ***p < .001, permutations = 999).

Abbreviations of the CSR plant life strategies: C, competitive; S, stress tolerating; R, ruderal. Abbreviations of the life cycle: A, annual; P, perennial.

Concerning the life cycle of indicator species, annuals prevailed on the LG sites. Perennials were present on the HG sites of the arid region and dominated in the semiarid region, on both HG and LG sites (Table 3).

There were not many indicator species in the open plots, all of them annual/perennial stress‐tolerant species (e.g., *Poa bulbosa* in HG sites of the arid region; *Aegilops triuncialis* and *Achillea biberesteinii* in LG sites of the semiarid region; Table 3).

## 4 DISCUSSION

### 4.1 Shrub canopy‐mediated abiotic and biotic stresses

Changes in relative interaction intensity (RII) indicate changes in interaction type along a combined gradient of grazing and aridity. For all of the three RII indices (cover, richness, and Shannon diversity), there was a decreasing trend from the highest toward the lowest stress levels (Figure 3). The shrub (*A. kopetdaghensis*) showed facilitative effects, preserving species diversity and richness and the total cover of species under its canopy. However, the facilitative effect was significantly stronger in the drier climatic region. Previous researchers (Bertness & Callaway, 1994; Brooker & Callaghan, 1998; Butterfield et al., 2016) have documented increases in the facilitation effect of the shrub by moderating the aridity stress. In arid environments, facilitation usually involves increasing the water and nutrient availability (Holzapfel & Mahall, 1999). Besides that, the shade from the shrub reduces extreme temperatures and decreases evaporation from the soil, which may further facilitate the germination of seeds and growth of seedlings. Therefore, this may explain why the shrubs show higher facilitation in the arid than in the semiarid regions (Farzam & Ejtehadi, 2017; Smit et al., 2007; Tirado et al., 2015).

The effect of *A. kopetdaghensis* canopy was consistently facilitative under intensive grazing. As *A. kopetdaghensis* is unpalatable, it is not usually grazed by livestock during the growing season. Therefore, it provides mechanical refugee for palatable grasses and forbs (reviewed by Baraza et al., 2006; Graff et al., 2007; Holthuijzen & Veblen, 2015; Milchunas & Noy‐Meir, 2002). This result is consistent with the “repellent plant hypothesis,” suggesting that grazing‐intolerant plants are protected by the surrounding grazing tolerant plants (Milchunas & Noy‐Meir, 2002).

### 4.2 Relative interaction index along the stress gradients

Various results have been reported, and some researchers indicated that the amelioration of abiotic stress was more important than protection from grazing (Arroyo et al., 2015; Howard et al., 2012). On the contrary, other studies demonstrated that grazing was a more important driver of the plant–plant interactions than abiotic stress in the African savanna (Filazzola et al., 2017; Louthan et al., 2014).

In the arid region, strong facilitation was observed in both grazing intensities, suggesting that the protection from aridity is more important than protection from intensive grazing (Maestre et al., 2005; Soliveres et al., 2011). Accordingly, a theory by Smit et al. (2009) predicts relatively low importance of protection from grazing in water‐limited environments. In arid climates, herbivores are sparsely distributed, and the availability of water or nutrients is more critical for vegetation than for protection from grazing (Ellis & Swift, 1988). In water‐limited environments, the shrubs usually improve soil fertility and microclimate under their canopies (Cortina & Maestre, 2005, Maestre et al., 2009). Also, shade from shrubs' and trees' canopy can retain soil moisture at the soil surface and facilitate neighbors with shallower roots (Maestre et al., 2003). Therefore, the dominant shrub may promote species richness and productivity by providing safe microsites for species growing in harsh conditions (Bruno et al., 2003).

On the other hand, in the semiarid region, where plants presumably grow in higher water availability, livestock grazing played a critical role in determining the type and relative intensity of the shrub's interaction with undercanopy species. The effect of the shrub's canopy (RII) was positive on the HG sites, but changed to negative with lower livestock grazing intensity. In harsh conditions such as high grazing intensity, the positive RII means the shrub can directly enhance survival rate, growth, and reproduction of other species by providing a more suitable environment under its canopy. However in low grazing intensity, a negative RII means that herbs prefer to grow in the open areas rather than under the canopy of shrubs, where they need to compete for light, nutrients, and water (Graff et al., 2007; Le Bagousse‐Pinguet et al., 2012).

### 4.3 Indicator species response to plant interactions in the condition of stress

This study shows that co‐occurring plant species under the shrub canopy may exhibit convergence in CSR plant life strategies in the conditions of similar levels of stress, while different stress levels lead to functional divergence. For instance, in the arid region, the dominant strategy of indicator species under *A. kopetdaghensis* converged to SR in the HG site. At the same time, species under the shrub's canopy exhibited transition from SR to R‐selected in low grazing intensity. S‐selected species prevail under the canopy of Artemisia, likely because important drivers of vegetation structure, such as disturbance (grazing) and stress (aridity), cause the loss of biomass (Caccianiga et al., 2006). However, on the LG sites in the arid region, the canopy protects the surrounding plants from aridity only, so the stress is less intensive than on the HG site. Therefore, most of the indicator species under the shrub's canopy were annual forbs and grasses with R strategy on the LG site. Stress‐intolerant species were better candidates for facilitation than stress‐tolerant species (Graff & Aguiar, 2011). For instance, in the arid conditions of Mediterranean shrublands, the stress‐avoidant species, with high specific leaf area and rapid growth, coexist with species featured by very low specific leaf area (Gross et al., 2013).

In the semiarid region, *A. kopetdaghensis* canopy showed a facilitative effect on the HG sites, supporting the establishment of species with C‐selected strategy, such as *Elymus hispidus* or *Lactuca orientalis*, which have larger leaves and are generally more palatable to livestock (Tajali, 2012). This is mainly because *A. kopetdaghensis* is an unpalatable, stress‐tolerant shrub, and its canopy creates microsites, protecting other species against grazing by large herbivores. In contrast, *A. kopetdaghensis* canopy has a competitive effect on the perennial stress‐tolerant species on the LG sites in the semiarid region. *A. kopetdaghensis* shrubs have to compete with the understory species for light and nutrients. Therefore, the dominant strategy of indicator species under *A. kopetdaghensis* in HG site shifted from C‐ to S‐selected in LG site.

We found only a few indicator species in the open plots in both the arid and semiarid regions. *Poa bulbusa* was present in the HG sites of the arid region, and *Aegilops triuncialis* and *Achillea biberesteinii* were on the LG sites of the semiarid region (Table 3). As suggested by Grime (1977), when the disturbance is relatively low, species with S‐strategy can maintain their dominance in a community by occupying aboveground and belowground space rather than by competing for resources.

## 5 CONCLUSIONS

Our results document that local‐scale biotic processes, such as facilitation by the shrubs, are important determinants of diversity patterns. In general, shrubs are known to provide refugee for species in harsh conditions, such as high aridity or overgrazing. Furthermore, we argue that the discrepancy in the literature on changes in plant–plant interactions may be partially explained by differences in plant life strategies of species in the conditions of the combined effect of biotic (grazing) and abiotic (aridity) stress. Therefore, in the arid region, drought‐escaping species such as ephemerals and ruderals (R‐selected) and species tolerating stress (S‐selected) but avoiding herbivory are highly dependent on the facilitation under the canopy of shrubs. However, in sites without severe aridity, canopy of the target shrub protected competitive species (C‐selected) in the conditions of high grazing intensity (low aridity/high grazing). On the contrary, on sites without intensive grazing and severe aridity (low aridity/low grazing), facilitative effects of the shrub turned to competitive. Restoration approaches are urgently needed, especially for dry rangelands, degraded by intensive grazing in countries that have limited resources. Understanding the role of plant–plant interactions can significantly contribute to designing a sustainable management of both arid and intensively grazed areas.

## CONFLICT OF INTEREST

None declared.

## AUTHOR CONTRIBUTIONS

**Soroor Rahmanian:** Conceptualization (equal); data curation (equal); methodology (equal); software (equal); writing–original draft (equal); writing–review and editing (equal). **Hamid Ejtehadi:** Conceptualization (equal); project administration (equal); supervision (equal); writing–review and editing (equal). **Mohammad Farzam:** Conceptualization (equal); funding acquisition (equal); project administration (equal); supervision (equal); writing–review and editing (equal). **Martin Hejda:** Conceptualization (equal); data curation (equal); methodology (equal); writing–review and editing (equal). **Farshid Memariani:** Data curation (equal); writing–review and editing (equal). **Petr Pyšek:** Investigation (equal); writing–review and editing (equal).

## Data Availability

Data will be made available in the Dryad Digital Repository (https://doi.org/10.5061/dryad.79cnp5hw8).

## APPENDIX A

Mean values of the relative interaction intensity (RII), corresponding to the relative effect of the canopy of *Artemisia kopetdaghensis* on the under‐canopy communities

**Table:**

Climatic region	Grazing	RII‐Richness	RII‐Cover	RII‐Shannon diversity
Arid	HG	0.2	0.33	0.16
	LG	0.19	0.26	0.18
Semi‐arid	HG	0.18	0.3	0.01
	LG	−0.14	−0.38	−0.11
